# There is a specific response to pH by isolates of *Haemophilus influenzae* and this has a direct influence on biofilm formation

**DOI:** 10.1186/1471-2180-14-47

**Published:** 2014-02-21

**Authors:** Nadiah Ishak, Alexandra Tikhomirova, Stephen J Bent, Garth D Ehrlich, Fen Z Hu, Stephen P Kidd

**Affiliations:** 1 Research Centre for Infectious Diseases, The University of Adelaide, North Terrace Campus, Adelaide, South Australia 5005, Australia; 2 School of Molecular and Biomedical Sciences, The University of Adelaide, North Terrace Campus, Adelaide, South Australia 5005, Australia; 3 Center for Genomic Sciences, Institute of Molecular Medicine and Infectious Disease, Philadelphia, PA 1902, USA; 4 Center for Advanced Microbial Processing, Institute of Molecular Medicine and Infectious Disease, Philadelphia, PA 1902, USA; 5 Department of Microbiology and Immunology, Drexel University College of Medicine, Philadelphia, PA 19102, USA; 6 Department of Otolaryngology-Head and Neck Surgery, Drexel University College of Medicine, Philadelphia, PA 19102, USA

**Keywords:** Biofilm, *H. influenzae*, pH stress, Transcriptomics

## Abstract

**Background:**

*Haemophilus influenzae* colonizes the nasopharynx as a commensal. Strain-specific factors allow some strains to migrate to particular anatomical niches, such as the middle ear, bronchi or blood, and induce disease by surviving within the conditions present at these sites in the body. It is established that *H. influenzae* colonization and in some cases survival is highly dependent on their ability to form a biofilm. Biofilm formation is a key trait in the development of chronic infection by certain isolates. This is exemplified by the contrast between the biofilm-forming strains found in middle ear infections and those isolates that survive within the blood and are rarely associated with biofilm development.

**Results:**

Screening a group of *H. influenzae* strains revealed only slight variations in their growth across a range of pH conditions. However, some isolates responded to a pH of 8.0 by the formation of a biofilm. While the type b capsular blood isolate Eagan did not form a biofilm and grew at the same rate regardless of pH 6.8-8.0, transcriptomic analyses demonstrated that at pH 8.0 it uniquely induced a gluconate-uptake and metabolism pathway, which concurrently imports H^+^. A non-typeable *H. influenzae*, isolated from the middle ear, induced biofilm formation at pH 8.0, and at this pH it induced a series of iron acquisition genes, consistent with previous studies linking iron homeostasis to biofilm lifestyle.

**Conclusions:**

Different strains of *H. influenzae* cope with changes in environmental factors using strain-specific mechanisms. These pathways define the scope and mode of niche-survival for an isolate. The pH is a property that is different from the middle ear (at least pH 8.0) compared to other sites that *H. influenzae* can colonize and infect. The transcriptional response to increasing pH by *H. influenzae* varies between strains, and pH is linked to pathways that allow strains to either continue free-living growth or induction of a biofilm. We showed that a biofilm-forming isolate induced iron metabolism pathways, whereas a strain that does not form biofilm at increasing pH induced mechanisms for growth and pH homeostasis based on sugar acid transport.

## Background

*Haemophilus influenzae* is a γ-Proteobacterium from within the order the Pasteurellacae. It is an obligate human commensal of the nasopharynx and in most cases it remains as a commensal but some strains can transit from the nasopharynx to other parts of the body and in doing so cause numerous types of disease [1]. There are strain-specific factors that enable pathogenic strains to transit to, and then survive within, different parts of the body, where the stresses of multiple environmental conditions require a breadth of adaptive abilities that permit survival and growth [2]. There are a number of physical parameters that are known to vary between parts of the human host, including: oxygen tension, carbon/energy/nitrogen source, pH and the presence of reactive oxygen and reactive nitrogen species. Defence against these can be directly encoded through detoxification genetic pathways, but also through broader mechanisms for environmental adaptation. In addition to specific pathways that respond to and deal with each of the damaging physical or chemical stressors present within the various environments the bacteria may encounter, many bacteria have a capacity to switch their lifestyle such that these stresses no longer cause damage to their cell. One key lifestyle adaptation is the switch from planktonic (or “free-living”) cells to a biofilm [3-5]. This process is based upon numerous features of the bacterial cell including alterations in their metabolism and physiology, the presence and nature of surface structures, and the general physical properties of the bacterial cell. The process of biofilm formation is defined in stages and each of these has specific features and profiles [2]. Put simply, under stressed conditions bacterial cells can switch from a free-living and a rapidly dividing phenotype to an altered metabolic form associated with cell-cell aggregation and attachment to a surface. There are then early, mid, and late stages for the maturation of a bacterial biofilm. The particular stresses that induce a change in lifestyle and subsequently the process of biofilm formation are poorly defined for many pathogenic bacteria, however antibiotic usage is certainly one, nutrient starvation and oxidative stress are others [4]. These conditions or signals do seem to be specific for different species. Despite some previous disagreement about the ability of *H. influenzae* to form a biofilm [6], there is now overwhelming evidence that *H. influenzae* use biofilm formation for survival within the host and certainly in their colonization of the host [7-13]. There are elements of *H. influenzae* which seem to be induced and therefore important for biofilm formation [13]. There are numerous examples of studies that have shown that iron uptake is central to growth within a biofilm [14-20].

There is a need to further characterise the differences between biofilm-forming and non-biofilm-forming isolates of *H. influenzae*. This can be accomplished through a comparison of the genetic and transcriptomic differences between *H. influenzae* strains that respond to stresses by forming a biofilm, and those that continue to grow under those conditions without forming a biofilm. Changes in pH provides a suitable stressor, being central to its colonisation of different anatomical niches, and identification of the molecular pathways that vary between such isolates would be significant in our understanding of *H. influenzae* pathogenesis.

*H. influenzae* strains and isolates display more variation than many other pathogens and underpinning the basis for the strain-specific actors that underlie their biofilm formation (recently reviewed [21,22]). Indeed, coupled to this, there are many features of the *H. influenzae* physiology [23-25] and stress response [26-30] that indicate that this particular host-adapted bacterium has unique molecular mechanisms for survival in the various locations of its host that it can exist.

The pH is known to be elevated in the middle ear, compared to other parts of the body [31,32] and in this niche there is some evidence that it is pH that induces particular isolates of *H. influenzae* to form a biofilm [33]. We have assessed the response of different clinical isolates of *H. influenzae* to changing pH; their growth and biofilm formation.

## Results and discussion

### The growth of different strains of *H. influenzae* with changing pH

The growth of 11 strains (Additional file 1: Table S1) of *H. influenzae* were assessed over a range of pH values; pH 6.8, 7.4 and 8.0 as the physiological pH is known to vary among host organs, tissues and niches. Even within a particular body site there can be spatial and temporal changes in pH as a consequence of specific events [31]. Despite this uncertainty in the precise nature of the pH value associated with host-pathogen microenvironments, it is clear that there are distinct differences between the primary site of colonization (nasopharynx) and the various sites of infection, including the lower respiratory tract, the blood and the middle ear. As an example, the blood can be 6.8-7.4 and the middle ear is usually considered to be around pH 8.0 [31,32]. We assessed pH response of a small set of isolates of *H. influenzae* that were known to colonise either the blood or the middle ear. We grew the bacteria (in liquid cultures, see Methods) at pH 6.8, 7.4 and 8.0 and plotted their growth curves (Additional file 1: Figure S1) and from this we calculated mean growth rates (Table 1 and Additional file 1: Figure S2). There were no clear patterns, and the observed changes represented only slight variations. The equivocal differences in growth at different pH levels does not exclude the possibility that the cells are responding differently, such as with an alternative lifestyle (biofilm formation).

**Table 1:** **Growth rates of *H. influenzae *** **isolates grown at different pH**

***Strain***	***Type***	***pH 6.8***	***pH 7.0***	***pH 8.0***
Rd KW20	Serotype d, non-capsular	0.414 ± 0.08*	0.515 ± 0.10	0.443 ± 0.12
86-028NP	NTHi, OM	0.330 ± 0.09	0.483 ± 0.05	0.435 ± 0.04
R2846	NTHi, OM	0.405 ± 0.11	0.587 ± 0.04	0.477 ± 0.09
NTHi-1	NTHi, lung	0.412 ± 0.07	0.243 ± 0.01	0.410 ± 0.08
R2866	NTHi, blood	0.291 ± 0.04	0.194 ± 0.01	0.300 ± 0.05
285	NTHi, OM	0.293 ± 0.05	0.367 ± 0.07	0.422 ± 0.10
C486	NTHi, OM	0.480 ± 0.03	0.446 ± 0.04	0.554 ± 0.05
Hi667	NTHi, OM	0.281 ± 0.04	0.338 ± 0.01	0.234 ± 0.02
Eagan	Serotype b, CSF	0.358 ± 0.03	0.386 ± 0.07	0.391 ± 0.08
R3264	NTHi, middle ear of healthy child	0.256 ± 0.04	0.303 ± 0.03	0.236 ± 0.06
86-66MEE	NTHi, OM	0.295 ± 0.04	0.258 ± 0.02	0.200 ± 0.04

*doubling per hour.

### The formation of biofilm by *H. influenzae* as a consequence of changing pH

Given that colonization by *H. influenzae* within various host niches, such as the middle ear, is linked to their induction of a biofilm, and increased pH is characteristic of these environments, we assessed the possibility that biofilm induction is a consequence of increased pH. It has been previously suggested that for *H. influenzae* the biofilm formation is induced at pH 8.0 [33]. We assayed for biofilm formation at pH 6.8, 7.4 and 8.0 (Additional file 1: Figure S3). These screening assays were performed by counting planktonic cells (as described in Methods) followed by washing and then releasing the biofilm cells, and counting colonies (to calculate colony forming units per mL; CFU/ml). In some strains, such as isolate R3264, there was significant induction of biofilm at pH 8.0 (Additional file 1: Figure S3). Other strains, including Eagan, did not form biofilm at any pH.

**Figure 1:**
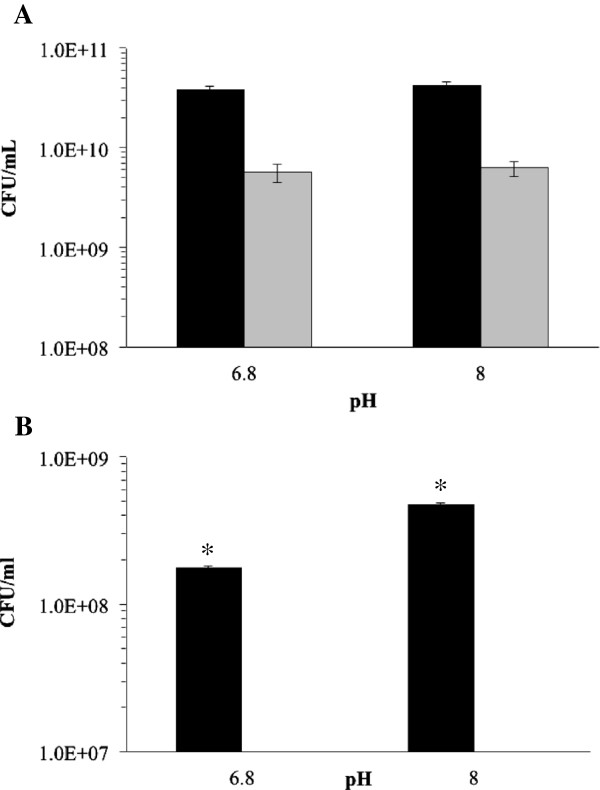
**The effect of pH on the (A) growth and (B) biofilm formed by *H. influenzae *isolates Eagan and R3264.** The cells of strain R3264 (black bars) and Eagan (grey bars) from planktonic **(A)** growth at pH 6.8 and then 8.0 were assessed. Similarly, the **(B)** biofilm cells were collected and cell numbers enumerated. Error bars are the standard deviation, *p < 0.001 (Student t-test).

To compare in detail contrasting isolates from this screening of *H. influenzae*, Eagan (a capsular, blood isolate) and R3264 (a NTHi middle ear isolate) were taken for further analysis (Figure 1), more biological and experimental replicates. Planktonic cell growth was assessed and then biofilm cell numbers were enumerated. Eagan grew equally well at pH 6.8 and 8.0, as did R3264, but Eagan did not form any biofilm at either pH 6.8 or 8.0 whereas R3264 produced a significant biofilm at pH 8.0, within the context of this assay there was an increase in biofilm formation at pH 8.0 (Figure 1B). These results are consistent with what is generally accepted and known with regard to *H. influenzae* pathogenesis; that the capsular strains cope with increased pH by continuing planktonic growth while NTHi isolates that colonizes the middle ear switches to a biofilm mode of growth [3,5,34].

### Transcriptional analyses of Eagan and R3264 under different pH

**Figure 2:**
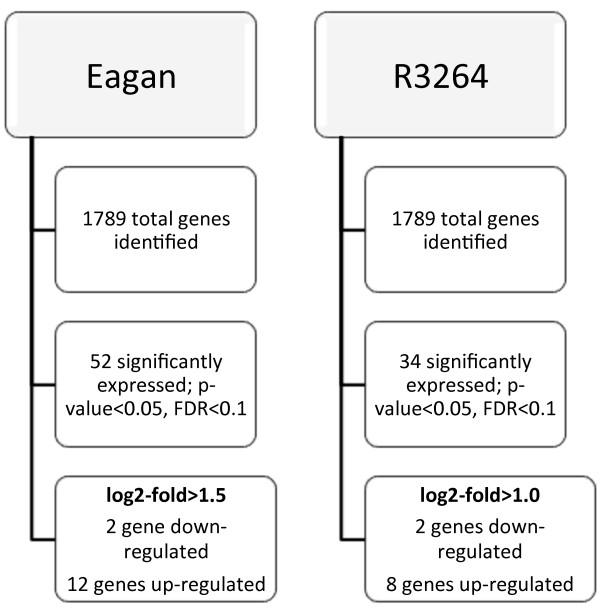
**An overview of RNAseq results for Eagan and R3264 growth at pH 6.8 and 8.0.** RNA was collected from planktonic growth of strains Eagan and R3264 when grown at pH 6.8 and 8.0 and the whole genome gene expression compared. The numbers of genes differentially expressed under these conditions is shown.

Given the definite, growth-style, variations in response to a shift in pH from 6.8 to 8.0 between Eagan and R3264, we were interested in determining the underlying transcriptional differences that varied between Eagan and R3264. We therefore used RNAseq to analyse the whole cell transcriptome at pH 6.8 and 8.0 for both Eagan and R3264 (Figure 2). The shift from pH 6.8 to 8.0, while biologically relevant and certainly impacting bacterial style of growth (Figure 2), is still a subtle change and it was not expected to generate a large set of cellular pathways with changed expression patterns.

**Figure 3:**
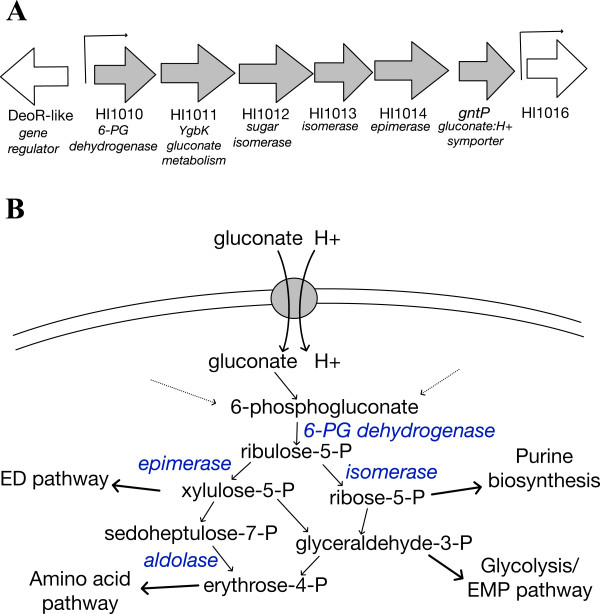
**The pathway uniquely induced in *H. influenzae *Eagan at pH 8.0. (A)** Genes HI1010-1015 (block arrows, grey) were all induced in *H. influenzae* Eagan at pH 8.0. *In silico* analysis identified 2 promoters across this region of the genome (indicated by line arrows) and HI1010-HI1015 forms a single operon. **(B)** These HI1010-1015 genes encode a gluconate:H^+^ symporter, a putative 6-phospohogluconate dehydrogenase and a range of sugar isomerases and epimerases that would link gluconate to the PPP and other metabolic pathways (the putative role for these genes are shown in blue).

Genes that were differentially expressed in Eagan (Table 2 and Additional file 1: Figure S4) revealed predominantly an up-regulation of two gluconate:H^+^ symporters (HI1015 and HI0092) and the associated gluconate (or sugar acid) metabolic genes (HI1010-1015, see Figure 3) and a potential glycerate kinase (HI0091) that links into glycolysis. It is worth noting that these genes/pathways are genetically unlinked, adding to validity of the response. In addition to the HI1015/*gntP* symporter, the HI1010-1015 genes include homologs to a sugar epimerase, aldolase and isomerases that are within the first stages of the pentose phosphate pathway (PPP). The first gene (HI1010) is a potential 6-phosphogluconate dehydrogenase that generates ribulose-5-phosphate. This links directly into the PPP and other energy and biosynthetic pathways (outlined in Figure 3).

**Table 2:** **Genes differentially expressed in *H. influenzae *** **Eagan at pH 8.0 compared to pH 6.8**

***Genes up-regulated at pH 8.0 compared to 6.8***				
**Metabolic genes**				
**Gene**	**Log**_**2**_** fold**	***p*-value**	**FDR**	**Comment**
HI1010	2.21	5.12×10^-10^	1.02×10^-7^	6-phosphogluconate dehydrogenase
HI1011	2.20	6.83×10^-10^	1.22x10^-7^	Similar to YgbK
HI1012	2.04	3.06×10^-8^	3.64x10^-6^	Sugar isomerase
HI1013	1.88	3.04×10^-7^	2.86×10^-5^	Hydroxypyruvate isomerase
HI1014	1.52	2.33×10^-5^	1.54×10^-3^	Sugar epimerase
HI1015	1.12	1.18×10^-3^	4.70×10^-2^	GntP family, gluconate:H^+^ symporter
HI0091	1.74	5.98×10^-7^	5.33×10^-5^	Hypothetical protein; homologous to GlxK, glycerate kinase
HI0092	2.14	1.49×10^-9^	2.41×10^-7^	GntP family, gluconate:H^+^ symporter
**Iron uptake genes**				
**Gene**	**Log**_**2 **_**fold**	***p*-value**	**FDR**	**Comment**
HI0995	1.53	1.72×10^-5^	1.23×10^-3^	OMP, iron-binding
*hitA*	2.21	1.69×10^-10^	3.77×10^-8^	Iron uptake
*hxuB*	1.65	1.54×10^-6^	1.25×10^-4^	Hemopexin utilization protein
*hxuC*	1.70	8.04×10^-7^	6.83×10^-5^	TonB-dependent heme receptor
**Genes of unknown function**				
**Gene**	**Log**_**2 **_ ***fold***	***p*-value**	**FDR**	**Comment**
HI1427	1.54	6.87×10^-6^	5.33×10^-4^	Hypothetical protein
***Genes down-regulated at pH 8.0 compared to 6.8***				
**Gene**	**Log**_**2 **_**fold**	***p*-value**	**FDR**	**Comment**
HI1349	-2.31	5.58×10^-11^	1.42×10^-8^	Ferritin
HI1385	-1.55	2.27×10^-5^	1.54×10^-3^	FtnB; non-heme ferritin

The GntP symporter family of transporters also import H^+^, as part of the survival response associated with an increased environmental pH (Table 2). It is interesting to note that our bioinformatic analyses have identified an operator/promoter upstream of HI1010 (Figure 3) with a putative DeoR binding site; HI1010 is divergent to a DeoR-like gene. While not within the scope of this project it is known in other bacteria that DeoR-like regulators variously control pathways directing sugar metabolism and are connected to the PPP. Also, the bioinformatics analyses indicate that the HI1010-1015 genes are on a single transcriptional unit, forming an operon.

Traditionally high concentrations of glucose are thought to be oxidized extracellularly by membrane-bound dehydrogenases. Whereas under low glucose conditions, oxidized glucose is imported and phosphorylated within the cell to 6-phosphogluconate [35] which feeds into either the Entner-Dourdooff (ED) pathway or the PPP [36] for energy. In addition, gluconate can act as an exogenous carbon source and therefore be taken up as a direct mode of growth. It has been shown in some contexts that such metabolism is related to bacterial growth in the host-pathogen environment, such as with *Escherichia coli* colonization of the mouse large intestine [37,38] where gluconate is also important in the growth and pathogenesis of other pathogens [39]. Some bacteria possess multiple gluconate uptake systems [40,41], such as those characterized in *E. coli*, where there are four [42]. Not all of these are necessarily primary gluconate transporters, with some acting on other sugar acids that are able to be utilized by the same permeases. At least one of these has been shown to be likely to preferentially import fructuronate and not gluconate [43]. In *E. coli* and other bacteria these transporters are regulated through different transcriptional pathways controlled by sugar-utilizing systems and signals; such as the sensing of the presence of gluconate by GntR, or as in a cAMP-dependent catabolite repression system/s, by the global transcriptional regulator CRP [40,44,45]. There is an emerging consensus that the regulation and role of these sugar acid metabolic systems is broader than originally thought. Recently it has been shown that in *E. coli*, the hexuronate utilizing pathways are regulated by a complex interplay of regulatory systems including induction under osmotic stress conditions [46]. What is clear from our results is that there are two homologous gluconate transport systems in *H. influenzae* Eagan and that both are upregulated at pH 8.0. The media used throughout our studies was rich in glucose and other carbon and energy sources (and the media was the same between pH 6.8 and 8.0; changes in carbon availability and the subsequent regulatory systems is therefore not a reason for these genes being upregulated at pH 8.0 compared to 6.8). It is worth noting that there are other genes responsible for these steps in the PPP in the genomes of *H. influenzae*, however these genes are not physically linked on an operon as with HI1010-1015. The indication is that in the Eagan strain the HI1010-1015 operon is uniquely regulated based on pH and it feeds into the PPP functioning under increased pH. The duplication of genes for steps in the PPP is not unusual, there are homologs of these *H. influenzae* genes (HI1011-1015) in several bacteria that have a similar duplication. In *Pectobacterium carotovorum* the homologs to HI1011-1015 are *vguABCD* and these function in gluconate metabolism and have an as yet uncharacterized role in the pathogenesis of this plant pathogen [47].

Interestingly, the sugar acid metabolism pathways can also feed into cell wall composition or modifications. Glucuronic acid can be incorporated into the inner core of the lipopolysaccharide (LPS) in *E. coli* and these potential modifications are a response to environmental stresses, specifically those associated with envelope stress, such as pH, and this response is controlled by several regulatory pathways [46,48].

We demonstrated that as the pH increases to 8.0, the Eagan isolate induced two gluconate permeases, one being part of an operon with gluconate metabolism genes, these likely providing the proteins and enzymes linked into energy production (through the ED or PPP pathways) but also potentially providing other cellular alterations for coping with the stress (modifying the LOS, for instance).

In contrast, the NTHi R3264 isolate did not induce the HI1010-1015 operon at pH 8.0. Consistent with this isolate inducing its biofilm formation at pH 8.0, it induced various, genetically unlinked iron acquisition genes (Table 3; the iron uptake genes *hitAB*, *tbp1-tbp2* and *hxuB* were all upregulated and the iron storage ferritin gene was down-regulated). In multiple bacterial species iron acquisition pathways have been linked to the development of the biofilm lifestyle; such that if these pathways are removed or iron is unavailable it depletes their biofilm-forming ability [16,19]. Likewise in studies on NTHi biofilm formation and biofilm maturation, the iron uptake has been shown to be essential [17,49-54]. It should be noted that in our comparative analyses of R3264 and Eagan at pH 8.0 we showed that Eagan did not form significant amounts of biofilm. As a comparison of their profile of growth pathways at pH 8.0 and then for R3264 at 6.8 (when R3264 cells forms less biofilm), the transcriptional switch in the planktonic R3264 cells at pH 8.0 compared to 6.8 is an indication of their response to this environmental condition and mechanisms that predispose the cells to biofilm formation as well as allowing a direct comparison to the Eagan planktonic cells at pH 8.0. The R3264 cells at pH 8.0 that are in the biofilm were therefore excluded from our comparison; these by definition would be greatly different (probably including the type IV pili or other adhesins) and not a clear comparison to the non-biofilm forming Eagan cells. It was not our aim to compare planktonic against biofilm cell but the response to increased pH, conditions we know shift the R3264 cells to biofilm-forming state. It is worth noting that there were iron-associated genes up-regulated in Eagan at pH 8.0 but not to the extent observed in R3264.

**Table 3:** **Genes differentially expressed in *H. influenzae *** **R3264 at pH 8.0 compared to pH 6.8**

***Genes up-regulated at pH 8.0 compared to 6.8***				
**Iron uptake genes**				
**Gene**	**Log**_**2 **_**fold**	***p*-value**	**FDR**	**Comment**
*hitA*	1.76	9.65×10^-12^	2.46×10^-9^	Iron uptake ABC, periplasmic domain
*hitB*	1.31	8.77×10^-7^	1.11×10^-4^	Iron uptake ABC, permease domain
*tbp2*	1.54	2.92×10^-5^	2.74×10^-3^	Iron-binding OM receptor
*tbp1*	1.49	3.53×10^-7^	5.26×10^-5^	Transferrin binding protein
*h×uB*	1.02	8.62×10^-5^	7.32×10^-3^	Heme-hemopexin utilization protein
**Metabolic genes**				
**Gene**	**Log**_**2 **_**fold**	***p*-value**	**FDR**	**Comment**
*glpB*	1.08	5.35×10^-5^	4.77×10^-3^	Glycerol metabolism
**Genes of unknown function**				
**Gene**	**Log**_**2 **_**fold**	*p***-value**	**FDR**	**Comment**
HI0997	1.34	8.95×10^-4^	5.51×10^-2^	Hypothetical protein
HI1427	1.31	4.17×10^-7^	5.72×10^-5^	Transmembrane protein
***Genes down-regulated at pH 8.0 compared to 6.8***				
**Gene**	**Log**_**2 **_**fold**	***p*-value**	**FDR**	**Comment**
HI1349	-1.23	5.14×10^-6^	5.10×10^-4^	Ferritin
*ahpD*	-1.72	1.24×10^-7^	2.01×10^-5^	Stress response

## Conclusions

*H. influenzae* can adapt to the physical and chemical properties that exist in different anatomical niches (such as the nasopharynx, lung, blood and the middle ear mucosa). Various strains of this pathogen adapt to these niches differently, such growing rapidly and planktonically or alternatively by forming a biofilm. The different niches are known to vary in a range of properties, the pH being one of these that subtly but significantly shifts from about neutral in the blood to pH 8.0 in the middle ear [31,32]. The pH does not remain constant within a niche and even in the blood there can various reasons for the pH to shift. While blood pH is tightly regulated at around pH 7.4, there are other parts of the body encountered by *H. influenzae* as a result of systemic infection starting in the blood that can include conditions that do reach pH 8.0. A capsular isolate taken from the blood would therefore need to be able to exist in the pH range of 6.8-8.0 but in this lifestyle it is rarely associated with a biofilm. A NTHi isolate from the middle ear (R3264) would predominantly encounter pH 8.0 and its processes of colonization would occur at this pH (although once again the pH is thought not to be constant in this niche, but varying within a range of 7.0-9.0). In this niche as part of its colonization, the bacterial cell would form a biofilm. Indeed some studies have shown that biofilm is induced in the middle ear as a very likely consequence of the increased pH (this was presented as a function of the induction of type IV pili but does not exclude other pathways not examined in this study) [33]. The type IV pili genes are more likely to be highly regulated in the biofilm cells themselves and not the planktonic cells we analysed.

Not all *H. influenzae* isolates respond to the changes in physical and chemical properties between the niches that *H. influenzae* can occupy with the same capacity or in the same manner. We show that *H. influenzae* isolates respond differently to the subtle and yet physiologically relevant changes in pH from 6.8 to 8.0. These changes are slight in regards to the observed growth rates but the changes are underpinned by lifestyle changes, such as modes of growth or biofilm formation. A capsular isolate (Eagan), continues to grow, with variation from pH 6.8 to 8.0 and does not form a biofilm while a NTHi isolate known to colonize the middle ear, does form a biofilm at pH 8.0. This is consistent with the established knowledge that the middle ear is more basic in its pH than the nasopharynx or the blood and NTHi colonize the middle ear by biofilm formation. While it was not unexpected that the NTHi isolate induced its iron-uptake pathways during its growth at pH 8.0 as it cells become predisposed to forming a biofilm, it was a novel finding that the Eagan strain induced gluconate:H^+^ uptake and sugar acid/gluconate metabolic genes. This pathway was not induced in the biofilm-forming R3264 cells. This obviously provides a pathway for growth, through the link from gluconate to the ED and PPP energy production pathways, while at the same time providing a mechanism for maintaining pH homeostasis (importing H^+^). Our study has therefore identified clear differences between a capsular isolate and a NTHi isolate in their response to a relevant pH shift; these differences seem likely to be the basis for their mode of growth and survival within a specific niche.

## Methods

### Bacterial strains and culture conditions

*H. influenzae* was cultured in BHI media which was prepared with 3.7% w/v BHI Powder (Oxoid). For solid media, 1.5% agar powder was added. Media was sterilized by autoclaving at 121°C for 20 minutes. 10% w/v Levinthal blood was added for solid BHI media. BHI broth required NAD^+^ (2 μg/ml) and 10 μl/ml Hemin solution (0.1% w/v Hemin, 0.1% w/v L-histidine, 4% v/v Triethanolamine). For monitoring cell growth over a time course, *H. influenzae* strains were initially cultured overnight in 5 ml BHI. The OD_600nm_ was measured and a normalized number of cells were inoculated into 250 μl of BHI broth in a 96-well plate (Falcon). The cells were grown with shaking, at 37°C in a incubating microtitre plate reader (BioTek, Es260). OD_600nm_ measurements were taken at given at 30 min. timepoints and the assays were performed in triplicate.

### Bacterial biofilm assays and assessment planktonic and biofilm cell numbers

In the first instance, the ability to form a biofilm was measured on polystyrene surfaces using 96-well plates (Microtest U-bottom, polystyrene, non-tissue culture treated plates, Falcon). Briefly, cells were grown for 24 hr at 37°C in the conditions as described for each experiment. The unattached cells were washed away with sterile water and the bound cells were stained with 0.1% crystal violet (at 4°C for 1 hr). The crystal violet was removed and the bound cells quantified by resuspending the crystal violet by addition of 250 μL 20% acetone: 80% ethanol and measuring the absorbance at 560 nm. Each sample had at least 4 replicates.

To concurrently assess planktonic and biofilm cells colony forming units per mL (CFU/mL) bacteria from each growth state were measured. Cells were grown as described above and then enumerated during the planktonic growth lifestyle: 20 μL are taken from 96-well plate growth, from the free-living broth culture. The 20 μL was added into 180 μL of PBS into a new 96-well plate. Serial dilutions were then performed from 10^-1^to 10^-8^ and 20 μL from each dilution was plated out onto Brain Heart Infusion (BHI) agar and incubated overnight in a 37°C/5% CO_2_ in incubator. Colonies were counted and CFU/mL calculated (CFU/mL = (number of colonies × 10^D^)/0.02). The values were plotted from the average of the samples with the error bars representing the standard deviation of the data. Samples were assayed in triplicate. For cells from the biofilm lifestyle; using the same plate as for the planktonic CFU/mL assay, the residual liquid was drained and the attached cells were washed three times with 200 μL of LB broth. After washing, 100 μL of fresh BHI media broth added into each well. The cells are detached by sonication for 3 seconds (Soniclean sonicating waterbath, a protocol established to disrupt bacterial attachment and aggregation), followed by removal of 20 μL from each well and a serial dilutions from 10^-1^ to 10^-8^ and plating onto BHI agar plates. Biofilm cells grow with an altered metabolism and it should be noted that the colonies on the plate appear different (generally smaller), but colony numbers are representative of live cell numbers within the system. CFU/mL are once again calculated using the formula; CFU/mL = (number of colonies × 10^D^)/0.02. The values were plotted from the average of the samples and the error bars represented the standard deviation of the data.

### Transcriptomic analysis

The selected strains; R3264 and Eagan were grown until late log-phase (16 hours) in 10 mL BHI liquid media and then cultured in BHI media broth in pH 6.8 and 8.0 for 3.5 hours before the collecting the cells for RNA extraction. To prevent RNA from degradation and preserved the RNA within the cells, cells were directly added to Phenol/Ethanol solution. The composition of phenol/ethanol solution is; 5% v/v Phenol (pH 4.3) and 95% v/v ethanol. The ratio used is 2/5 of the total cell culture volume: phenol/ethanol. This was left on ice for 2 hours before being centrifuged for 5 min. (4˚C/4000×g) and the supernatant discarded. The cell pellet was kept at -80˚C until RNA extraction. RNA is extracted using RNAeasy Mini kit according to RNAeasy mini standard protocol (QIAGEN). The RNA quality of the samples were checked with the Agilent Bioanalyzer (according to Agilent RNA 6000 Nano kit standard protocol; samples were loaded into RNA Nano chip and run using Agilent 2100 Bioanalyser machine). For each sample three biological replicates of cell growth, harvesting and RNA extraction was performed. The RNA was pooled.

RNA was provided to the Adelaide Cancer Genomic Research Facility (Adelaide Australia) for library preparation and sequencing (RNAseq) using the Ion Proton platform (Life Technologies).

The analysis pipeline used Bowtie2 [55] align reads from both samples to the *H. influenzae* RdKW20 reference genome (Genbank: NC_000907), followed by processing with SAMtools and BEDTools to generate a mapped read count for the reference genes from each sample. Differential expression analysis was performed using R program within the package edgeR and DESeq. In R, the genes that are statistically significant using the Benjamini-Hochberg procedure with a false discovery rate controlled at <0.1 are recorded in the Results section.

## Competing interests

The authors declare that they have no competing interests.

## Authors’ contributions

The research project was devised by SJB and SPK. Assays were undertaken and methodology refined by NI and AT, data were analysed by NI, AT SJB, GDE, FZH and SPK. The manuscript was written by NI, SJB and SPK and edited by GDE and FZH. All authors read and approved the final manuscript.

## Supplementary Material

Additional file 1: Table S1 Summary information of the strains used in this study. **Figure S1.** The growth profile of different strains of *H. influenzae* grown under different pH. **Figure S2.** The growth rates of *H. influenzae* strains under different pH. **Figure S3.** Viable cell counts of different *H. influenzae* strains grown under pH 6.8, 7.4 and 8.0. **Figure S4.** Scatter plots of log2 fold change against normalized counts for each of the genes identified from mRNAseq. Click here for file
