# Supplementary material for: There is a specific response to pH by isolates of Haemophilus influenzae and this has a direct influence on biofilm formation
Source: BMC Microbiol. 2014 Feb 21;14:47. doi: 10.1186/1471-2180-14-47 (PMC3938079; doi:10.1186/1471-2180-14-47)
Supplement: Additional file 1: Table S1 — Summary information of the strains used in this study. Figure S1. The growth profile of different strains of H. influenzae grown under different pH. Figure S2. The growth rates of H. influenzae strains under different pH. Figure S3. Viable cell counts of different H. influenzae strains grown under pH 6.8, 7.4 and 8.0. Figure S4. Scatter plots of log2 fold change against normalized counts for each of the genes identified from mRNAseq. [file 1471-2180-14-47-S1.pdf]

Table S1: Summary information of the strains used in this study.

Strain	Genotype	Source isolated
Rd KW20	Serotype d, non - capsular	Lab strain
285	non-typeable (NTHi)	Otitis Media
86-028NP	NTHi	Otitis Media
**86.0276MEE**	NTHi	Otitis Media
**C486**	NTHI	Otitis Media
Eagan	Serotype b, capsular	Cerebral Serum Fluid
Hi667	NTHi	Otitis Media
**R2846**	NTHi	Otitis Media
R2866	NTHi	Blood
R3264	NTHi	Middle ear of Healthy Child
**165**	NTHi	Otitis Media

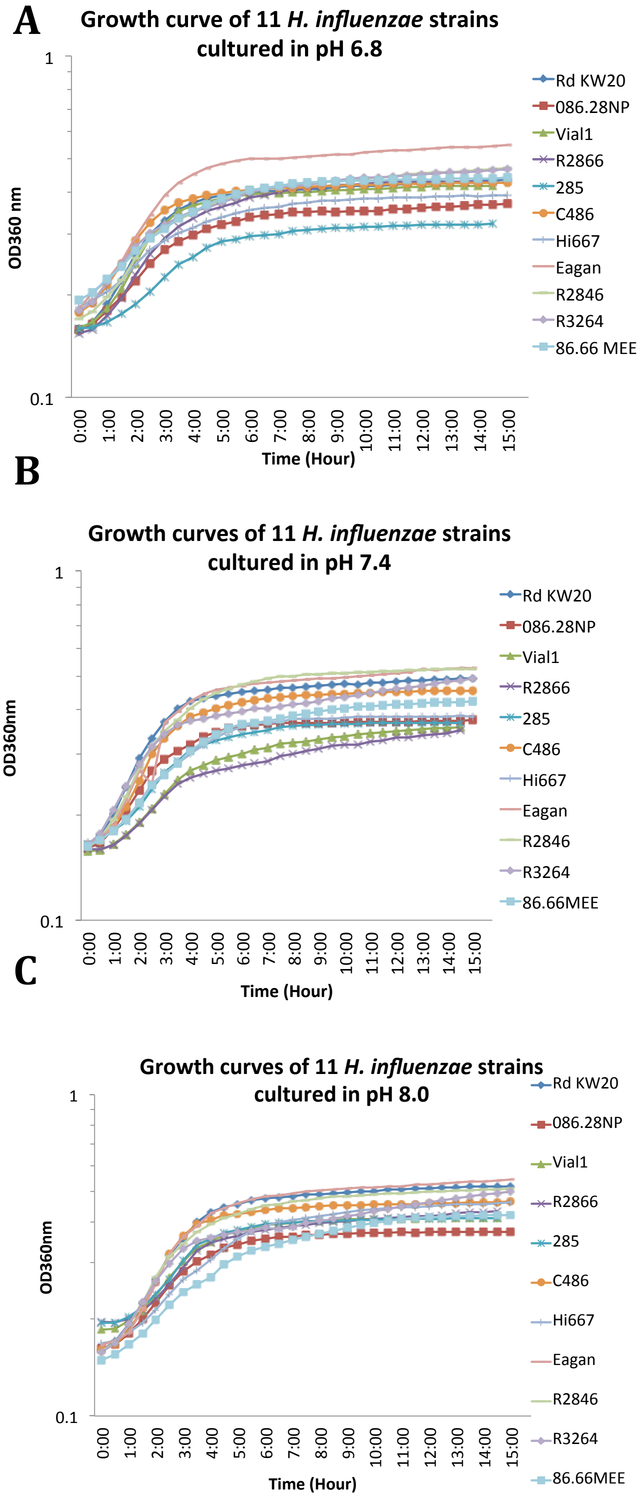

Figure S1: The growth profile of different strains of *H. influenzae* grown under different pH. The *H. influenzae* isolates as indicated were grown in HI broth pre-culture before a normalized amount inoculated into media at (A) pH 6.8, (B) pH 7.4 and (C) pH 8.0.

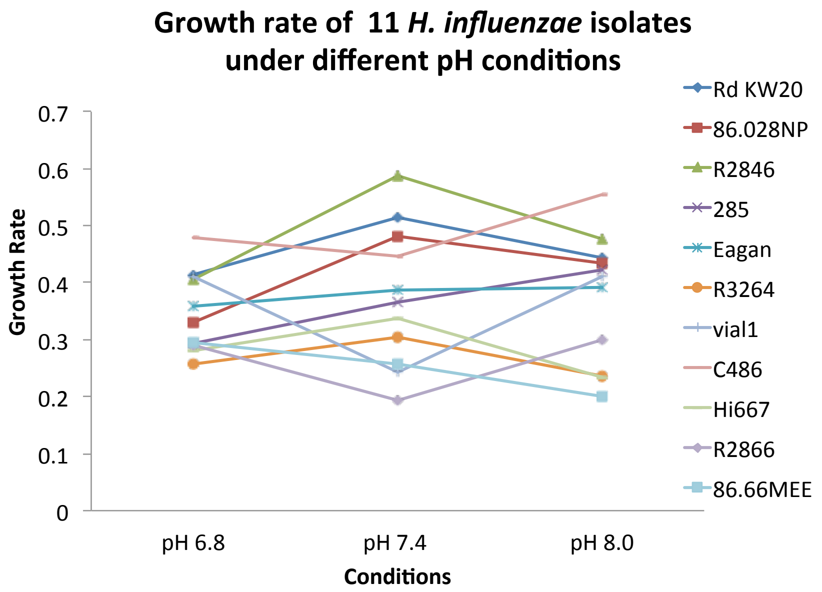

Figure S2: The growth rates of *H. influenzae* strains under different pH. The growth rates were calculated from Fig S1 and plotted.

A

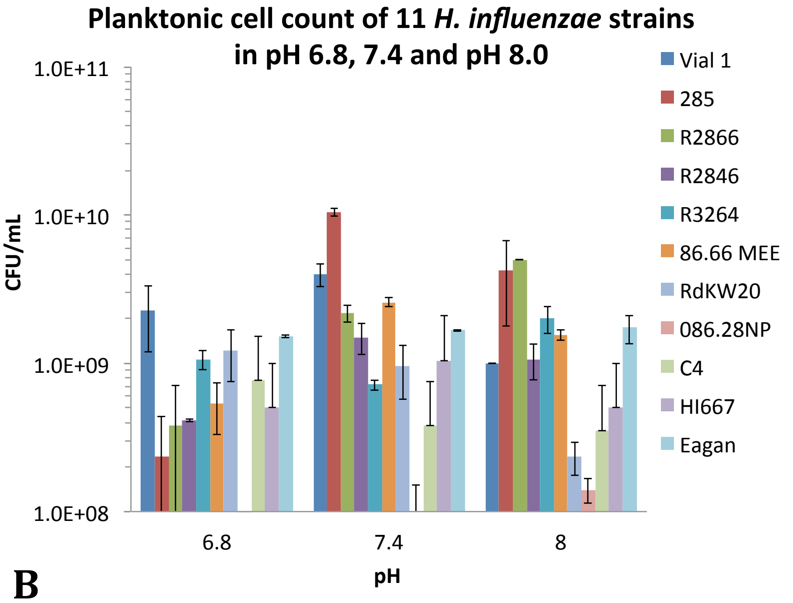

B

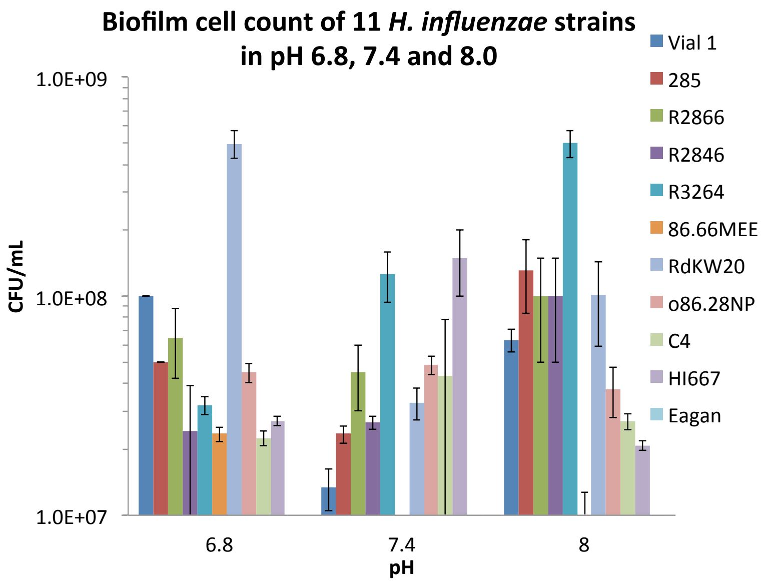

Figure S3: Viable cell counts of different *H. influenzae* strains grown under pH 6.8, 7.4 and 8.0. The isolates were grown and viable cells counted for the (A) planktonic cell number (CFU/ml) and concurrently the (B) biofilm cell number.

A

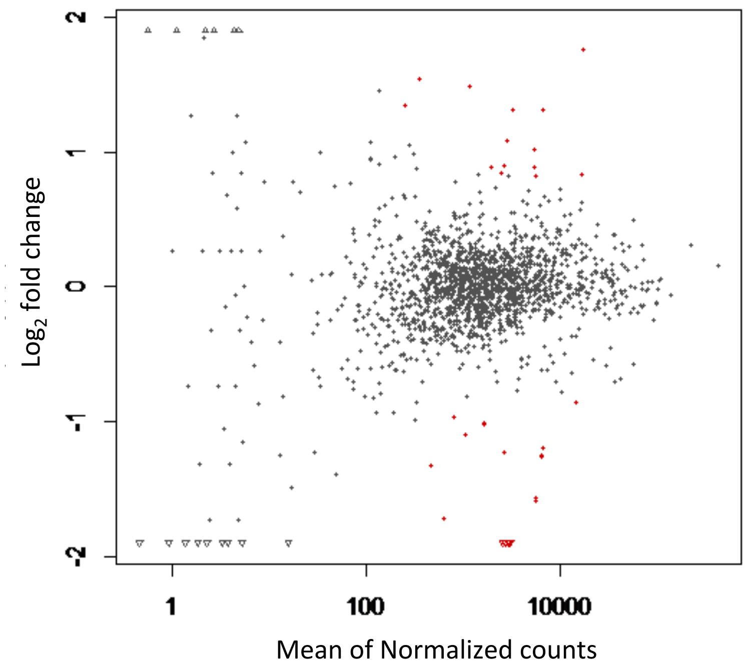

B

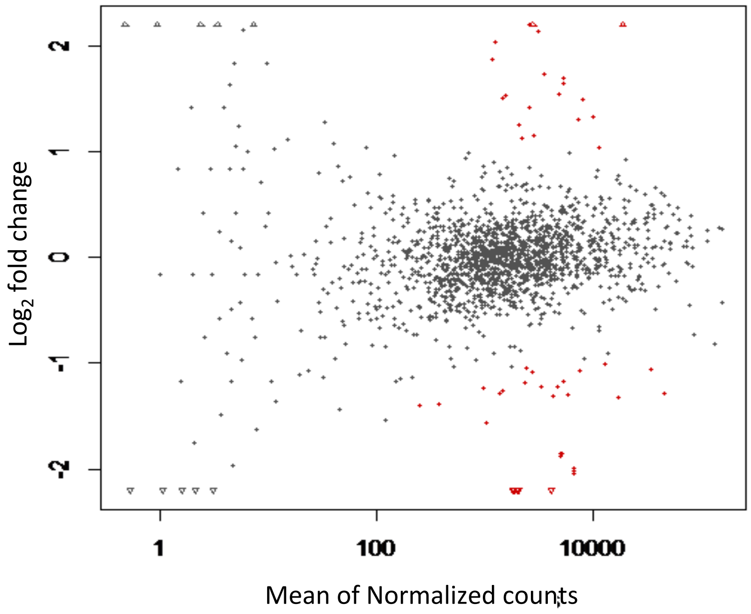

Figure S4: Scatter plots of $\log_2$ fold change against normalized counts for each of the genes identified from mRNAseq. Each dot represents a gene identified from the RNA sequencing library and then compared for counts in the library from pH 8.0 compared to pH 6.8 for (A) R3264 and then for (B) Eagan. The dots that red are those genes determined as being significantly differentially expressed with p-value<0.05 and FDR<10%.
